# Residential mobility in pregnancy and potential exposure misclassification of air pollution, temperature, and greenness

**DOI:** 10.1097/EE9.0000000000000273

**Published:** 2023-10-26

**Authors:** Seulkee Heo, Yelena Afanasyeva, Leonardo Trasande, Michelle L. Bell, Akhgar Ghassabian

**Affiliations:** a School of the Environment, Yale University, New Haven, Connecticut; b Department of Population Health at NYU Grossman School of Medicine, New York University, New York, New York; c Department of Pediatrics at NYU Grossman School of Medicine, New York University, New York, New York

**Keywords:** Fetal development, Air pollution, Maternal exposure, Environmental exposure, Birth cohort, Greenspace

## Abstract

**Introduction:**

Epidemiological studies commonly use residential addresses at birth to estimate exposures throughout pregnancy, ignoring residential mobility. Lack of consideration for residential mobility during pregnancy might lead to exposure misclassification that should be addressed in environmental epidemiology.

**Methods:**

We investigated potential exposure misclassification from estimating exposure during pregnancy by residence at delivery utilizing a prospective cohort of pregnant women in New York, United States (n = 1899; 2016–2019). We calculated exposure during pregnancy corresponding to each address for fine particles (PM_2.5_), temperature, and greenness (Enhanced Vegetation Index [EVI]).

**Results:**

Twenty-two percent of participants moved at least once during pregnancy; 82.3% of movers changed residences during the second or third trimesters. Participants with better health, lower parity, and higher socioeconomic status were more likely to move. Exposures based on address at delivery rather than residential history overestimated exposure for PM_2.5_ (exposure error: range −5.7 to 4.6 µg/m^3^, average −0.6 µg/m^3^) and EVI (range −0.305 to 0.307, average −0.013), but not temperature. Overestimations were significantly larger for mothers with higher socioeconomic status. Our findings indicate that the error for prenatal exposure can occur when residential mobility is not considered and is disproportional by maternal characteristics.

**Conclusions:**

Epidemiological studies should consider residential mobility in exposure assessments based on geolocation when possible, and results based on mother’s residence at birth should be interpreted with understanding of potential differential exposure misclassification.

**What this study adds**

This study is among the first to report empirical evidence on the degree of errors in environment exposure during pregnancy due to neglecting residential mobility of pregnant women. Our study findings suggest that residential mobility patterns differ by socioeconomic status of pregnant women and so the resultant errors in exposure measurement to particulate matter and residential greenness. This study highlights needs for future epidemiological studies to consider residential mobility in exposure assessment during pregnancy.

## Introduction

Developing fetuses are vulnerable to adverse effects of environmental exposure due to the rapid prenatal development of the brain and other organs.^1^ The fetal period is a vital exposure window where developmental damages can lead to lifelong health consequences.^2^ Prenatal exposure to environmental factors, such as air pollution and ambient heat (e.g., heatwave), is associated with increased risks of impaired fetal growth (e.g., low birth weight, small for gestational age), preterm birth, stillbirth, and birth defects.^3,4^ A growing body of literature suggests protective effects of residential greenness (i.e., the amount of vegetation or vegetated area around homes) for birth weight through several potential pathways: reduced exposure to environmental toxicants, stress relief, and increased physical activity.^5^ Climate change is projected to significantly increase population exposure to heatwaves and air pollution leading to increased risks of adverse birth outcomes globally, and such risks will be amplified for susceptible groups such as socioeconomically deprived mothers.^6^

Most epidemiologic studies of adverse birth outcomes in relation to geospatially-variable environmental exposures use residential address at delivery to estimate exposure levels during pregnancy^5,7–11^ as information on residential mobility during pregnancy is usually unavailable from many relevant health datasets, such as birth certificate data. The degree to which environmental exposure estimates are affected by residential mobility during pregnancy may result in exposure misclassification that could obscure important environmental health associations, although evidence on this exposure misclassification is limited.^12^ Further, if such exposure misclassification from residential mobility varies by mothers’ individual-level factors such as socioeconomic status, results on differences in effect estimates by those subgroups could be biased. For example, in a recent systematic review of environmental studies in the United States, more than three-quarters of the birth outcome studies relied on maternal residential address at delivery to assess prenatal environmental exposures.^3^ This type of exposure assessment assumes that women do not move during pregnancy or that residential mobility during pregnancy has negligible impacts on estimates of exposure.

If the exposure-outcome associations are associated with maternal factors that are also related to residential mobility during pregnancy and early infancy, the errors in exposure could influence the risk estimates for the subgroups that are potentially more susceptible to misclassification for prenatal exposure.^12^ Several studies have investigated characteristics of individuals who changed residences during pregnancy. A US case-control study based on the Texas Birth Defects Registry found that about 33% of women moved, with lower mobility during pregnancy for women with higher income and Hispanic ethnicity.^7^ A review study on residential mobility during pregnancy found that the percentage of mobility was between 9% and 32% in the 14 identified studies.^13^ Although informative, these studies mostly collected information on residential mobility retrospectively after delivery.

Investigations based on birth cohorts may include information on maternal residential mobility throughout pregnancy. Although full residential histories may be unclear as residences are often recorded at specific follow-up time points (e.g., prenatal care visit), information on the residential history during pregnancy recorded in a prospective design provides an opportunity to examine potential exposure misclassifications resulting from using the address at delivery as a proxy of spatial environmental exposure. Moves with higher distances would likely contribute to different exposure levels for environmental factors, although moves of short distances could also introduce exposure misclassification for exposures with high spatial heterogeneity. Records of residences during pregnancy can help examine the degree of misclassification by maternal individual-level factors.

This study examined the influence of residential mobility during pregnancy on exposure estimates of environmental factors using data from a birth cohort in New York City, United States. We investigated: (1) individual-level maternal and/or paternal factors associated with moves during pregnancy, and (2) differences in the environmental exposures during pregnancy estimated based on address at delivery and those estimated considering residential mobility with all available addresses during pregnancy. We focused on air pollution, temperature, and residential greenness as exposure of interest since these factors have been widely studied for their associations with adverse birth outcomes.^14,15^

## Methods

### Study participants

The NYU Children’s Health and Environment Study (CHES) was initiated to answer multidisciplinary research questions on the impact of preconceptual, prenatal, and postnatal environmental exposures on children’s health including neurodevelopment, growth, and respiratory outcomes.^16^ The NYU CHES is a clinically enrolled prospective cohort in three diverse hospitals serving a wide array of populations in New York: NYU Langone—Manhattan, NYU Langone—Brooklyn, and Bellevue Hospital. Eligible pregnant women were ≥18 years old, <18 weeks pregnant, had a pregnancy that was not medically threatened, and planned to deliver at one of the study hospitals (enrollment between 2016 and 2019). Prenatal data collection through questionnaires (eAppendix; http://links.lww.com/EE/A242) and specimen collection were conducted during three routine prenatal visits (<18, 18–25, and >25 weeks of gestation).

The NYU CHES cohort recruited 2193 pregnancies (2000 live births) between March 2016 and April 2019, with substantial racial/ethnic and socioeconomic diversity.^16^ About 49% of the participants were Hispanic, 65% were employed, and 48% had private insurance. Most participants were married (88%) and about half (51%) had a bachelor’s degree or higher educational attainment. Our analysis included mothers of singleton live births, who provided one or more residential addresses during pregnancy. All participating women provided written informed consent; the Institutional Review Board of the New York University Grossman School of Medicine approved the study.

### Covariates

We considered several maternal individual-level factors suggested to be associated with residential mobility of pregnancy women by previous studies^13^: age at enrollment, pre-pregnancy weight and height (which was used to calculate body mass index), marital status, educational attainment (high school or less, some college but no degree, associate degree, bachelor’s degree, and post-graduate degree), household income (<$30,000, $35,000–74,000, $50,000–74,000, $75,000–99,000, ≥$100000), employment status (employed, unemployed), alcohol usage before/during pregnancy, use of nicotine or marijuana during pregnancy, self-reported health status (poor, fair, good, very good, excellent), physical activity during pregnancy (quantiles of the average score of the International Physical Activity Questionnaire-Short Form across all three trimesters), insurance type (public vs. private), birth country (born in or outside the United States), mother’s race/ethnicity (Asian, non-Hispanic [NH] White, NH Black, Hispanic, multi-race, other). We also considered father’s race/ethnicity, educational level, and age.

### Residential mobility

Residential addresses were obtained from self-administered questionnaires at the three routine prenatal visits in three pregnancy intervals (<18 weeks; 18–25 weeks; >25 weeks) and at birth. We also extracted addresses from electronic health records for the period 3 months before pregnancy start date and through delivery. Each residential address was geocoded using DeGAUSS.^17,18^ The period of residence at one address was calculated between the date at which that address was recorded and the day before the date of recording at the next address. We defined participants who moved to the address at delivery during pregnancy as a “mover” in this analysis. Participants without records of addresses other than multiple identical records matching the address at delivery during pregnancy were defined as “non-movers.” For each mover, we examined in which trimester they moved. The total distance moved for each mover was calculated using the ArcMap software.

### Exposure assessment

We calculated environmental exposures during pregnancy for each participant in two ways: (1) average exposure levels corresponding to the time and location of residential addresses throughout the pregnancy and (2) average exposure levels for duration of pregnancy based on residence at delivery. For air pollution, we consider PM_2.5_, which is often investigated in air pollution studies, has well-established harmful effects on human health, and is regulated by the US Environmental Protection Agency (EPA).^19^ PM_2.5_ has been also associated with adverse birth outcomes consistently among previous review studies.^3,20,21^ We linked each address to the nearest EPA monitoring station^22^ within 20 km. For the two metrics of exposure (i.e., based on all addresses during pregnancy vs. based on address at delivery) of daily PM_2.5_ observations were for each trimester and whole pregnancy, which are important exposure windows that are often considered in environmental birth outcome studies.^23^

For temperature, we used the gridded (1 × 1 km2 resolution) daily maximum temperature estimates for 2015–2019 provided by the Daymet dataset.^24^ The monthly average temperature in 2018 obtained from the Daymet was shown to present geographical variability of temperature in the study area (Figure S4; http://links.lww.com/EE/A242). The gridded estimates were averaged for pregnancy and each trimester for varying sizes of buffers for residential addresses (e.g., 100-, 250-, 500-, 1000-, and 2000-m). Greenspace-health associations may vary by the buffer sizes as different buffer sizes of residential greenness may have different health pathways (e.g., removing air pollution, promoting physical activities, and psychological effects). These buffer sizes were based on the commonly used buffer sizes of greenness in previous studies.^25–27^

Exposure to residential greenness was estimated using the Enhanced Vegetation Index (EVI), an advanced version of the Normalized Difference Vegetation Index, with corrections for distortions in the satellite sensor observations by aerosols, ground cover below vegetation, and chlorophyl.^28^ Both indices range from −1 to 1, with higher values indicating denser vegetation. We obtained 8-day composite images of EVI at 30 × 30 m^2^ resolution for 2015–2019 from the Landsat-7 Collection 1 Tier 1 database generated by the US Geological Survey (USGS) and archived in the Google Earth Engine. We calculated average EVI values for pregnancy and each trimester using EVI pixel values within 100-, 250-, 500-, 1000-, and 2000-m of mothers’ residential addresses. The main results of temperature and EVI were presented using estimates based on 100-m buffers.

### Statistical analysis

We used Chi-squared and Fisher’s tests to investigate patterns of residential mobility (i.e., moved vs. not moved) by health-related indicators (e.g., alcohol use, smoking status before and during pregnancy, mother’s general health status, mother’s age, mother’s physical activity during pregnancy, parity, marital status) and socioeconomic or other demographic indicators (race/ethnicity of mother and father, mother’s education, household income, insurance type, mother’s employment status, and mother’s birth country). The *t*-test and analysis of variance (ANOVA) compared the distances moved among the movers by these factors. We estimated the errors in exposure as the difference between the exposure based on address at delivery and exposure based on all residences throughout pregnancy, which more accurately reflects actual exposure. We used *t*-test and ANOVA to compare the degree of exposure errors by individual-level parental factors. Statistical analyses were conducted using R software version 4.2.2.^29^

## Results

**Figure 1.**
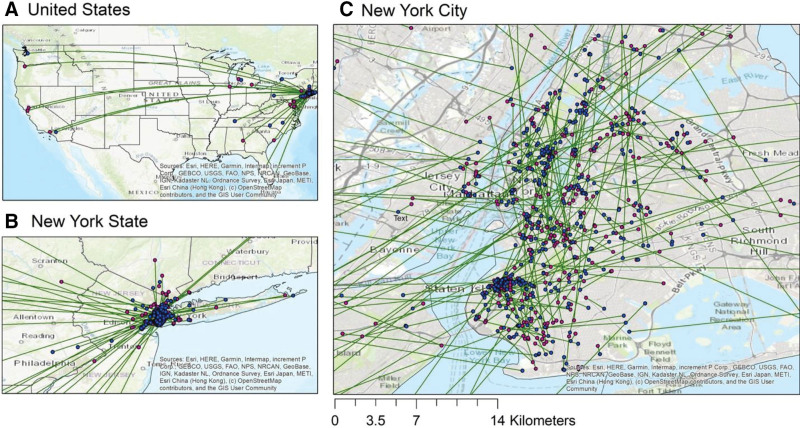
Residential mobility map: the link between the residence at delivery and the previous address for each mover. Blue dot: start point. Magenta dot: endpoint (i.e., address at delivery).

This study included 1,899 pregnant women with singleton livebirths in NYU CHES between 2016 and 2019. Study participants were predominantly Hispanic (48.2%) followed by non-Hispanic White (34.0%), had high income (39.4% with household income ≥$100,000 USD), and were married (88.1%) (Table S1; http://links.lww.com/EE/A242). Of the 1899 mothers, 417 (22.0%) moved at least once during pregnancy (Table 1). About 43.4% of the movers moved to the address at delivery during the third trimester, while 39.3% moved during the second trimester. The median moving distance was 2.8 km (range 0.002 km to 7736.4 km). About 61.4% of the movers moved <5 km in total. Figure 1 shows the linkage between the address at delivery and the previous address for each mover.

**Table 1.** Patterns of residential mobility.

	*N* (%)
Total	1899 (100.0)
Movers	417 (22.0)
Non-movers	1482 (78.0)
Frequency of moves (number of moves during pregnancy)	
1	316 (75.8)
2	81 (19.4)
3	16 (3.8)
4	4 (1.0)
Timing of move to address at delivery	
First trimester	72 (17.7)
Second trimester	164 (39.3)
Third trimester	43 (43.4)
Distance of moves (km, category)	
<5	256 (61.4)
5–9	56 (13.4)
10–19	36 (8.6)
≥20	69 (16.5)
Distance of movement (km, continuous)	
Min–max	0.002–7736.4
25th percentile	0.6
Median	2.8
Mean	143.5
75th percentile	10.0
Standard deviation	747.5

Table 2 shows the difference between movers and non-movers by individual-level factors. Participants who moved and non-movers had significantly different characteristics with respect to general health status, parity, and nativity. Also, participants who moved were more likely to have higher education, higher income, and private insurance than non-movers. Participants born in the United States were more likely to move at least once compared with those born elsewhere.

**Table 2.** Results of the chi-square test for individual-level factors and residential movement during pregnancy (*n* = 1899).

	Non-mover	Mover	*P*
Total	1482 (78.0)	417 (22.0)	-
Age at enrolment			
<20	35 (2.4)	6 (1.4)	0.20
20–24	153 (10.3)	51 (12.2)	
25–29	310 (20.9)	81 (19.4)	
30–34	536 (36.2)	172 (41.2)	
35–39	352 (23.8)	83 (19.9)	
≥40	96 (6.5)	24 (5.8)	
Age at enrolment, mean ± SD	32.4 ± 5.6	32.14 ± 5.3	<0.05
Race/ethnicity^b^			
Asian	126 (8.6)	50 (12.0)	0.09
Hispanic	733 (50.1)	181 (43.5)	
Multi-race	32 (2.2)	8 (1.9)	
NH-Black	80 (5.5)	26 (6.3)	
NH-White	493 (33.7)	151 (36.3)	
Education of mother			
High school of less	491 (33.1)	105 (25.2)	0.01
Some college but no degree	165 (11.1)	50 (12.0)	
Associate degree	77 (5.2)	16 (3.8)	
Bachelor’s degree	336 (22.7)	100 (24.0)	
Post-graduate degree	404 (27.3)	141 (33.8)	
Unknown	9 (0.6)	5 (1.2)	
Marital status			
Divorced/separated	31 (2.1)	9 (2.2)	0.20
Married/living with a partner	1296 (87.5)	377 (90.4)	
Single/widowed	154 (10.4)	31 (7.4)	
Annual household Income			
Less than $30,000	243 (16.4)	55 (13.2)	<0.001
$30,000–$49,999	137 (9.2)	23 (5.5)	
$50,000–$74,999	101 (6.8)	25 (6.0)	
$75,000–$99,999	68 (4.6)	20 (4.8)	
$100,000 or more	524 (35.4)	203 (48.7)	
Unknown	409 (27.6)	91 (21.8)	
Insurance type			
Public	790 (53.3)	168 (40.3)	<0.001
Private	680 (45.9)	247 (59.2)	
Unknown	12 (0.8)	2 (0.5)	
Employment			
Employed	520 (35.2)	130 (31.3)	0.15
Unemployed	957 (64.8)	286 (68.8)	
Nativity			
Born in the United States	644 (43.5)	211 (50.6)	0.01
Born outside the United States	687 (46.4)	178 (42.7)	
Unknown	151 (10.2)	28 (6.7)	
BMI, mean ± SD	26.4 ± 5.8	25.4 ± 5.0	<0.05
General health status of mother, %			
Poor or fair	49 (3.3)	12 (2.9)	0.01
Good	381 (25.7)	109 (26.1)	
Very good	588 (39.7)	192 (46.0)	
Excellent	302 (20.4)	81 (19.4)	
Unknown	162 (10.9)	23 (5.5)	
Alcohol use, %			
Never	501 (33.9)	123 (29.6)	0.05
Before pregnancy	764 (51.7)	215 (51.7)	
Before and during pregnancy	212 (14.4)	78 (18.7)	
Smoking, %			
Never	1343 (90.8)	380 (91.3)	0.97
Before pregnancy	120 (8.1)	32 (7.7)	
Before and during pregnancy	17 (1.1)	4 (1.0)	
Physical activity during pregnancy^a^			
Q1	342 (23.1)	104 (24.9)	0.65
Q2	358 (24.2)	103 (24.7)	
Q3	365 (24.6)	88 (21.1)	
Q4	350 (23.6)	104 (24.9)	
Unknown	67 (4.5)	18 (4.3)	
Parity			
Nulliparous	717 (48.4)	232 (55.6)	0.01
Parous	764 (51.6)	185 (44.4)	
Race/ethnicity of father^b^			
Asian	88 (9.0)	33 (10.9)	0.06
Hispanic	501 (51.3)	172 (56.6)	
Multi-race	283 (29.0)	63 (20.7)	
NH-Black	6 (0.6)	3 (1.0)	
NH-White	99 (10.1)	33 (10.9)	
Education of father			
High school of less	166 (11.2)	45 (10.8)	0.62
Some college but no degree	279 (18.8)	64 (15.3)	
Associate degree	119 (8.0)	32 (7.7)	
Bachelor’s degree	45 (3.0)	12 (2.9)	
Post-graduate degree	283 (19.1)	89 (21.3)	
Unknown	590 (39.8)	175 (42.0)	

a Higher scores for higher physical activities.

b Excluding “other” races due to small sample size (*n* < 2).

BMI indicates body mass index.

There were no significant differences in the total distance moved during pregnancy by participants’ characteristics except for insurance type (Figure S1; http://links.lww.com/EE/A242). The distance moved was significant higher for participants with private insurance (median 4.64 km, SD 958.2) than for participants with public insurance (median 1.52 km, SD 105.5) (*P* < 0.05). Among all participants, women with higher socioeconomic status (e.g., high education, high income, private insurance) were more likely to move compared with participants with lower socioeconomic status (Table 1). However, among movers, higher socioeconomic status was inversely related to frequency of moves during pregnancy. Among movers, the lower education level groups among the five categories (i.e., <high school, some college, associate degree, bachelor’s degree, and post-graduate degree) showed gradually higher frequencies of moves during pregnancy (*P =* 0.02). Participants with higher income (e.g., ≥$75,000 vs. <$75000) were more likely to have more moves during pregnancy (*P* = 0.01). While the total distance moved during pregnancy was on average higher for participants with private insurance, the frequency of moves was slightly lower for these women (average 1.2, SD 0.51) than participants with public insurance (average 1.33, SD 0.63) (*P* = 0.09).

The average, SD, and range of the differences of weekly gestational PM_2.5_ exposures based on residential histories and exposures based on address at delivery for 417 movers were −0.62, 4.3, and −19.6–166.5 µg/m^3^, respectively. The average difference in EVI was −0.061 and SD was 0.128 with a range of −0.725–0.560.

**Figure 2.**
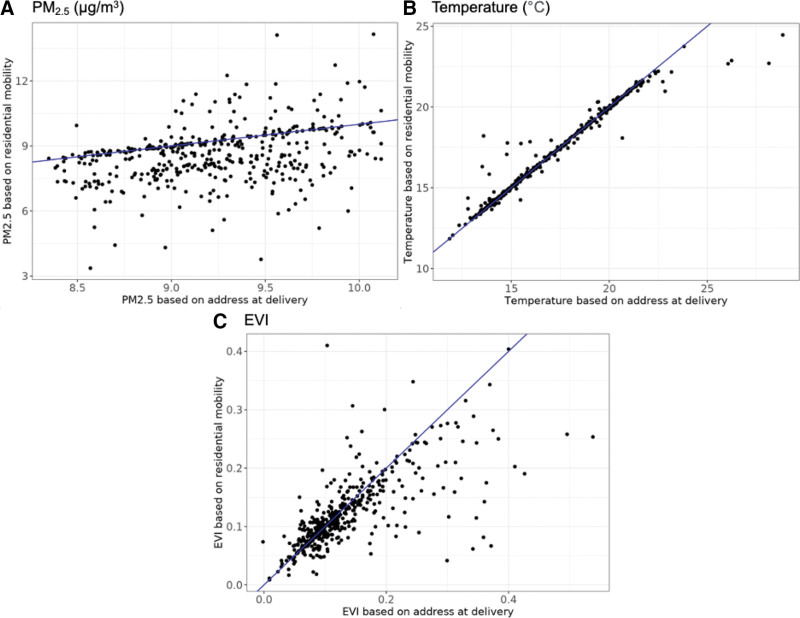
Scatter plot of exposure levels based on different exposure estimates: x-axis for exposures measured for the address at delivery; y-axis for exposures measured based on residential mobility during pregnancy (*n* = 417). The blue lines are at slope of 1, reflecting perfect agreement. A, PM_2.5_, (B) temperature within 100-m buffers, and (C) EVI within 100-m buffers.

Figure 2 shows compare exposure estimates based on address at delivery and those based on all residential addresses throughout pregnancy. PM_2.5_ based on residential mobility minus PM_2.5_ based on address at delivery ranged from −5.7 to 4.6 µg/m^3^ (average −0.6, SD 1.3). The difference in temperature (within 100-m buffers) ranged from −6.2 to 4.6 ^o^C (average 0.0, SD 0.7). The range of the difference for EVI (within 100-m buffers) was −0.305 to 0.307 (average −0.013, SD 0.058). Exposure misclassification for EVI and temperature did not vary substantially by buffer size used to assign exposure (Figure S2; http://links.lww.com/EE/A242).

**Figure 3.**
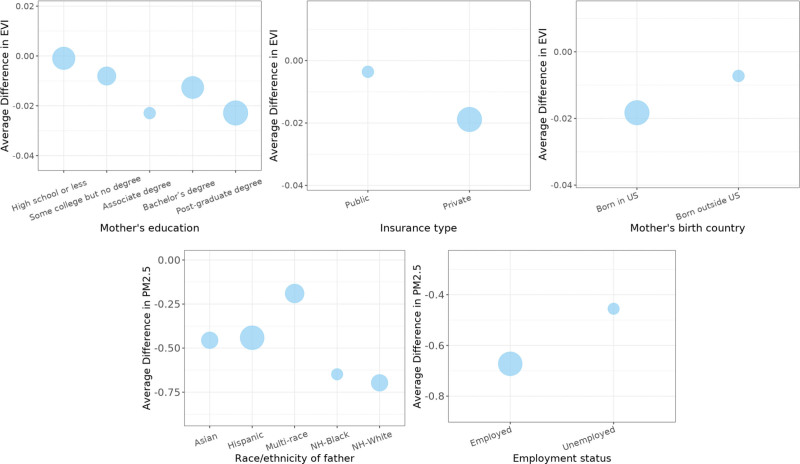
Difference between the exposure levels based on residential mobility and the exposure levels based on the address at delivery for EVI and PM_2.5_ concentration, by education, employment status, insurance type, and mother’s birth country. The size of the point reflects the percent of observations for that category of the x-axis.

The correlations between maternal/paternal factors and prenatal exposure based on full residential history versus address at delivery were examined (Figure 3 and Table S2; http://links.lww.com/EE/A242). There were significant overestimations in PM_2.5_ exposure based on address at delivery by race/ethnicity of the infant’s father. Overestimations in PM_2.5_ were higher for NH-Black and NH-White paternal race/ethnicity (*P* = 0.05). EVI was significantly higher when based on residence at delivery than residential history for participants with higher educational attainment (post-graduate degree vs. other education levels) (*P* < 0.05), with private insurance (*P* < 0.05), and infants born outside the United States (*P* = 0.06). There were no significant differences in temperature exposure by the examined covariates.

Factors associated with exposure errors differed by the exposure considered. Participants with higher education (post-graduate degree vs. other), those who were employed, and mothers born in the United States were more likely to have larger differences between EVI exposures based on residential history versus address at delivery (i.e., overestimation) (Figure 3). For PM_2.5_, the overestimation of exposure by ignoring the residential history was significantly higher for infants with paternal NH-Black and NH-White race/ethnicity and employed status (Figure 3). No factor in the analysis was significantly associated with exposure errors for temperature.

## Discussion

This study investigated individual characteristics associated with residential mobility in pregnant women participating in a birth cohort in the NYC metropolitan area. We examined the extent to which omitting residential mobility affects misclassifications for environmental exposures assessed using residential addresses. Overall, 22% of participants moved during pregnancy, which is within the range observed in previous studies using other datasets (9%–32%).^13^ Our results demonstrated that residential mobility of pregnant women was not random and errors for prenatal environmental exposures ignoring residential mobility were differential depending on several maternal/paternal factors including socioeconomic indicators. The factors associated with the degree of exposure errors differed by the type of environmental exposure considered.

The timing of residential mobility during pregnancy varied in previous studies. We found the lowest tendency to move in the first trimester, whereas some other studies, conducted in Atlanta, Georgia, and New York state, United States, found the lowest mobility in the third trimester.^30,31^ The majority of studies, including our study, found the highest mobility during the second trimester.^13^ Higher mobility earlier in pregnancy would contribute more substantially to exposure misclassifications as most environmental studies of fetal health and birth outcomes use address at delivery to estimate exposure.

Previous studies found that certain characteristics were associated with higher residential mobility during pregnancy. Generally, in previous studies using cohort, birth certificate, or birth defect surveillance system data, higher mobility was found for mothers who were younger, unmarried, had lower parity, had lower education, or had lower household income.^13^ However, we did not find significant differences in mobility by maternal age or marital status. We found higher mobility for women who were healthier, non-Hispanic, born in the United States and had higher education/income or private insurance. Inconsistent results for who is more likely to move during pregnancy across studies indicate that patterns of residential mobility differ by population.^13^ Furthermore, our results, showing that higher socioeconomic status was negatively associated with the number of moves during pregnancy but positively associated with distance moved, indicate the complexity of patterns of residential mobility and subsequent misclassification of environmental exposures. The findings for the number of moves by SES are important as more frequent moves of those with lower SES may indicate a struggle with affordability related to housing costs.

A few studies investigated the extent to which using address at delivery to estimate exposure contributes to biased estimates of prenatal exposure to environmental factors.^30,32,33^ Most studies did not find significant misclassification. Some studies presumed that exposure misclassification would not be greatly influenced by residential mobility if the distance moved was small. The degree to which moves within short distances will contribute to exposure misclassification will depend on the spatial patterns of exposures and spatial heterogeneity. About 61.4% of pregnant women moved <5 km during pregnancy in our study. Our study found that exposure misclassification differed by the exposure; we found misclassification by residential mobility for PM_2.5_ and residential greenness, but not temperature. While the average difference between the exposure based on residential mobility and the exposure based on the address at delivery for PM_2.5_ exposure during pregnancy was −0.6 µg/m^3^, the difference ranged from −5.7 to 4.6 µg/m^3^ implying a substantial misclassification for some participants. The degree of misclassification for EVI exposure was also substantial for some participants. The range of differences measured per gestational week was more pronounced: −15.0 to 84.1 µg/m^3^ for PM_2.5_ and −0.736 to 0.735 for EVI (Figure S3; http://links.lww.com/EE/A242).

Exposure misclassification was differential by participants’ characteristics. Infants’ health is associated with parental age, height, weight, and lifestyle.^34–37^ It is also potentially associated with the race or ethnicity of the mother, father, or both.^38^ Studies have shown that paternal race/ethnicity is an important predictor of low birth weight for infants of Caucasian and Hispanic mothers.^39^ We found that the overestimation in exposure of PM_2.5_ based on the address at delivery was on average higher for NH-White or employed mothers. On the other hand, the likelihood of overestimation for EVI was higher for mothers with higher educational attainment, those with private insurance, and those who were born in the United States. These results suggest that the degree of misclassification introduced by ignoring residential mobility may be larger for some demographic and/or socioeconomic subgroups, which is critically important for investigations of environmental health disparities. It also suggests that the individual-level factors contributing to exposure misclassification by residential mobility vary by the type of environmental exposure. Furthermore, this type of exposure error in continuous variables of exposure can lead to serious uncertainty of risk estimations for outcomes if their values are modified into a categorical variable using boundary cutoffs or the errors are nondifferential with respect to the outcome.^40^

Several studies examined associations between greenness on pregnancy outcomes by varying buffer sizes and suggested that associations were consistent among different buffer sizes.^41,42^ Some studies assumed that larger buffers around residences are more prone to exposure misclassification than smaller buffers. While future studies need to evaluate the optimal buffer size to use in health studies, the degrees of misclassification for EVI and temperature during pregnancy were similar across different buffer sizes in our study.

Some studies examined environmental exposure misclassifications by gestational weeks. Warren et al. examined errors for prenatal PM_10_ exposure by gestational week in Connecticut using birth cohorts, finding that exposure misclassification was more pronounced during earlier pregnancy weeks.^33^ However, we did not find such patterns by gestational week for exposure misclassification for PM_2.5_ or EVI, possibly due to differences in population characteristics and residential mobility. The percentage of women who moved at least once in the Connecticut birth cohorts was 10.97%, whereas over 20% of mothers in our data moved at least once during pregnancy. The median distance moved was 5 km in the Connecticut birth cohorts, but shorter (2.8 km) in our analysis. These results indicate that the impact of residential mobility during pregnancy on exposure misclassification might be differential by geographical regions and populations, warranting local population-based research on environmental exposure errors and birth outcome. Population-specific results are also important given that potential biological mechanisms and relevant exposure windows may vary by the type of birth outcomes.^43^

There are several strengths in this study. First, data on maternal and paternal factors were obtained prospectively during follow-up visits, lessening the probability of recall bias. Similarly, address histories were obtained prospectively. Many previous studies utilizing information on residential histories during pregnancy collected residential history after delivery possibly subject to recall bias.^13^ Also, this information is not available from other types of birth data such as birth certificate data. Second, our address history data had high spatial and temporal resolution, whereas some epidemiological studies using birth certificate data rely on residential ZIP codes or census tracts, potentially resulting in exposure misclassifications.^43^ Third, the cohort in our analysis was racially and ethnically diverse. Fourth, to our best knowledge, this is the first study using a prospective cohort study to examine errors in prenatal exposure to residential greenness by incomplete information on the residential mobility of pregnant women. Given the inconclusive study results for the impacts of prenatal greenness exposure on birth outcomes (e.g., low birth weight) among previous studies,^44^ using more precise environmental exposure estimates such as those investigated in our analysis would benefit future studies to better estimate the influence of greenspace on birth outcomes as the exposure of interest or as an effect modifier of other exposures.

This study also has limitations. We used monitoring data of PM_2.5_ for air pollution exposure due to the lack of data for our study period and regions. While this has the strength of using actual measurements, exposure estimates are related to the distribution of the monitoring network. Other air pollution data such as interpolated data or modeling data may lead to different results of the degree of measurement errors for PM_2.5_ exposure. Second, our cohort was racially and ethnically diverse, but our results have limited generalizability as we used the cohort data from the three study hospitals in the New York City area, and comparison of findings across studies indicates that patterns of residential mobility vary across populations. Third, we used complete residential histories collected during each visit by the participating mothers as the gold standard, but there may be possibilities of unrecorded residences within the data. This may be critical for identifying movers and calculating mobility rates in our data. Also, we defined the residency start date as the date the address was collected and there may be differences between the actual residency start date and our defined residency start date. We assumed that such differences would be minor throughout pregnancy. Fourth, we did not have information on the daily time-activity patterns of the participants, which is a source of uncertainty in exposure estimates. Daily time-activity pattern may be also different by demographic status. This may potentially lead to another exposure misclassification, although such type of misclassification was not main interests of this study. Last, while we examined various exposures associated with birth outcomes, it is unclear how the results for the degree of misclassifications would be robust depending on different data sources for the same exposure (e.g., different methods to estimate PM_2.5_ exposure). Misclassification is also a function of spatial variation of exposure factors and the size of geographic zones for exposure assignments.^30,45^ For example, our analysis found exposure misclassification in residential greenness using the satellite imagery data of EVI but results for errors may change under different estimates for spatial proximity to greenness (e.g., nearest park). The spatial resolution of the modeled data for temperature may affect detecting exposure errors for heat. Nonetheless, our study aim was to examine the impact of neglecting residential mobility during pregnancy for prenatal exposure estimates rather than to identify the best type of environmental data sources for avoiding exposure misclassifications. The impact of omitting residential mobility in exposure assessment for birth outcomes studies will likely differ by the type of environmental exposure and the way in which that exposure is assessed. Last, the number of movers in our analysis may be small for sub-group analyses by SES on exposure misclassification. The findings on SES factors without significant results on exposure misclassification due to ignoring residential mobility in our analyses may be related to limited statistical power from a small sample size. Nonetheless, our sample size was relatively higher than most of the previously published studies on residential mobility of pregnant women given that about 65% of the previous studies had a sample size of less than 1500 persons.^13^

Rises in ambient temperature under climate change and deteriorated air quality due to rapid global urbanization will have major implications for infants’ health, as will other changes such as the degree to which cities have greenspace. Accurate exposure assessment for environmental exposures is crucial for informing effective decision-making to prevent environmental risks to maternal and child health, especially to assess impacts on vulnerable populations. Our analysis using data on residential mobility of a New York cohort data found that the impact of residential mobility of pregnant women on exposure estimates is not random. Certain groups are more likely to move and there are differential misclassifications for prenatal exposure by the type of environmental exposure, with more misclassification for PM_2.5_ and residential greenness than temperature and with different degrees of misclassification by maternal/paternal characteristics. Future studies should examine how the degree of exposure misclassification differs among sub-populations and how such errors induce bias in critical exposure windows and the risk estimations for different types of birth outcomes, as well as different environmental exposures and methods of exposure assessment.

## Conflicts of interest statement

The authors declare that they have no conflicts of interest with regard to the content of this report.

## Supplementary Material
