# Supplementary material for: Residential mobility in pregnancy and potential exposure misclassification of air pollution, temperature, and greenness
Source: Environ Epidemiol. 2023 Oct 26;7(6):e273. doi: 10.1097/EE9.0000000000000273 (PMC11189681; doi:10.1097/EE9.0000000000000273)
Supplement: Supplementary file 1 [file ee9-7-e273-s001.docx]

Supplementary materials

Residential mobility in pregnancy and potential exposure misclassification of air pollution, temperature, and greenness

**Content**

Table S1. Baseline characteristics of the participating mothers (*n* = 1899).

Table S2. Statistical results for the average difference (standard deviation) between the exposure levels based on residential mobility and the exposure levels based on the address at delivery for PM_2.5_ concentration, temperature (100-m buffer), and EVI (100-m buffer) (n = 417).

Figure S1. Frequency of moves and the total distance moved during pregnancy among the mothers who moved, by maternal factors (n = 417), for factors for which movers and non-movers were statistically different.

Figure S2. Exposure misclassification for EVI by buffer size.

Figure S3. Exposure misclassification by gestational week.

Figure S4. Map of the monthly averages of daily maximum temperature for New York City in 2018 from the Daymet.

eAppendix - Data collection instruments

Table S1. Baseline characteristics of the participating mothers (*n* = 1899).

|  | *N* (%) |
| --- | --- |
| Age at enrollment (years) |  |
| <20 | 41 (2.2) |
| 20–24 | 204 (10.7) |
| 25–29 | 391 (20.6) |
| 30–34 | 708 (37.3) |
| 35–39 | 435 (22.9) |
| 40+ | 120 (6.3) |
| Age (mean, SD) | 31.9 (5.5) |
| Race/ethnicity of mother |  |
| Hispanic | 914 (48.2) |
| Non-Hispanic White | 644 (34.0) |
| Non-Hispanic Black | 106 (5.6) |
| Asian | 176 (9.3) |
| Other | 15 (0.8) |
| Multi-race | 40 (2.1) |
| Marital status |  |
| Married/living with a partner | 1673 (88.1) |
| Divorced/separated | 40 (2.1) |
| Single/widowed | 185 (9.7) |
| Education of mother |  |
| High school or less | 596 (31.6) |
| Some college but no degree | 215 (11.4) |
| Associate degree | 93 (4.9) |
| Bachelor’s degree | 436 (23.1) |
| Post-graduate degree | 545 (28.9) |
| Annual household income |  |
| Less than $30,000 | 298 (16.1) |
| $30,000–$49,999 | 160 (8.7) |
| $50,000–$74,999 | 126 (6.8) |
| $75,000–$99,999 | 88 (4.8) |
| $100,000 or more | 727 (39.4) |
| Unknown | 447 (24.2) |
| Insurance type |  |
| Public | 958 (50.8) |
| Private | 927 (49.2) |
| Employment status |  |
| Employed | 1243 (65.7) |
| Unemployed | 650 (34.3) |
| BMI (mean, SD) | 26.2 (5.7) |
| Recruitment hospitals |  |
| Bellevue | 345 (18.2) |
| NYU Brooklyn | 589 (31.0) |
| NYU Manhattan | 965 (50.8) |

Table S2. Statistical results for the average difference (standard deviation) between the exposure levels based on residential mobility and the exposure levels based on the address at delivery for PM_2.5_ concentration, temperature (100-m buffer), and EVI (100-m buffer) (*n* = 417).

|  | PM_2.5_ | *p*-value | Temperature | *p*-value | EVI | *p*-value |
| --- | --- | --- | --- | --- | --- | --- |
| Race/ethnicity of mother |  |  |  |  |  |  |
| Asian | -0.37 (1.29) | 0.435 | -0.02 (0.17) | 0.898 | -0.021 (0.055) | 0.19 |
| Hispanic | -0.59 (1.17) |  | -0.05 (0.70) |  | -0.007 (0.038) |  |
| Multi-race | -0.42 (1.29) |  | -0.06 (0.21) |  | 0.000 (0.131) |  |
| NH-Black | -0.91 (1.58) |  | -0.04 (0.59) |  | -0.005 (0.054) |  |
| NH-White | -0.66 (1.29) |  | 0.03 (0.78) |  | -0.018 (0.069) |  |
| Race/ethnicity of father |  |  |  |  |  |  |
| Asian | -0.27 (1.35) | 0.048 | -0.17 (0.96) | 0.292 | -0.016 (0.053) | 0.70 |
| Hispanic | -0.60 (1.29) |  | 0.05 (0.52) |  | -0.017 (0.068) |  |
| Multi-race | -0.43 (1.01) |  | -0.10 (0.82) |  | -0.004 (0.058) |  |
| NH-Black | -1.26 (1.22) |  | 0.02 (0.12) |  | -0.020 (0.034) |  |
| NH-White | -1.14 (1.70) |  | -0.07 (0.55) |  | -0.013 (0.046) |  |
| Marital status of mother |  |  |  |  |  |  |
| Divorced/separated | -1.16 (1.68) | 0.455 | 0.02 (0.07) | 0.266 | -0.012 (0.058) | 0.43 |
| Married/living with a partner | -0.58 (1.26) |  | -0.03 (0.65) |  | -0.025 (0.046) |  |
| Single/widowed | -0.69 (1.18) |  | 0.11 (0.99) |  | -0.019 (0.061) |  |
| Education of mother |  |  |  |  |  |  |
| High school of less | -0.53 (1.03) | 0.129 | 0.01 (0.13) | 0.495 | -0.001 (0.032) | <0.05 |
| Some college but no degree | -0.43 (1.45) |  | -0.31 (0.98) |  | -0.001 (0.042) |  |
| Associate degree | -0.48 (1.00) |  | 0.35 (1.16) |  | -0.023 (0.090) |  |
| Bachelor’s degree | -0.61 (1.15) |  | 0.03 (0.26) |  | -0.013 (0.047) |  |
| Post-graduate degree | -0.74 (1.45) |  | 0.00 (0.88) |  | -0.023 (0.077) |  |
| Education of father |  |  |  |  |  |  |
| High school of less | -0.46 (1.01) | 0.621 | 0.03 (0.17) | 0.683 | 0.001 (0.036) | 0.20 |
| Some college but no degree | -0.64 (1.09) |  | -0.04 (0.36) |  | -0.005 (0.041) |  |
| Associate degree | -0.38 (1.13) |  | 0.17 (0.82) |  | -0.019 (0.061) |  |
| Bachelor’s degree | -0.64 (1.65) |  | -0.37 (1.06) |  | -0.022 (0.083) |  |
| Post-graduate degree | -0.61 (1.33) |  | -0.04 (0.50) |  | -0.012 (0.063) |  |
| Income (annual) |  |  |  |  |  |  |
| Less than $30,000 | -0.22 (0.73) | 0.115 | 0.06 (0.74) | 0.900 | -0.014 (0.052) | 0.60 |
| $30,000–$49,999 | -0.47 (0.84) |  | -0.17 (0.93) |  | -0.007 (0.048) |  |
| $50,000–$74,999 | -1.13 (1.77) |  | -0.17 (0.76) |  | -0.019 (0.067) |  |
| $75,000–$99,999 | -0.51 (1.56) |  | -0.07 (0.34) |  | -0.002 (0.033) |  |
| $100,000 or more | -0.64 (1.34) |  | 0.00 (0.78) |  | -0.019 (0.070) |  |
| Unknown | -0.61 (1.14) |  | 0.00 (0.10) |  | 0.001 (0.022) |  |
| Insurance type |  |  |  |  |  |  |
| Public | -0.57 (1.09) | 0.597 | 0.02 (0.43) | 0.341 | -0.004 (0.036) | <0.05 |
| Private | -0.64 (1.35) |  | -0.04 (0.81) |  | -0.019 (0.068) |  |
| Employment status of mother |  |  |  |  |  |  |
| Employed | -0.67 (1.31) | 0.087 | -0.01 (0.79) | 0.570 | -0.015 (0.061) | 0.34 |
| Unemployed | -0.46 (1.14) |  | -0.04 (0.31) |  | -0.009 (0.051) |  |
| Mother’s birth country |  |  |  |  |  |  |
| Born in US | -0.67 (1.41) | 0.161 | -0.01 (0.81) | 0.556 | -0.018 (0.070) | 0.06 |
| Born outside US | -0.49 (1.08) |  | -0.05 (0.52) |  | -0.007 (0.045) |  |
| Parity |  |  |  |  |  |  |
| Nulliparous | -0.65 (1.19) | 0.392 | 0.03 (0.70) | 0.145 | -0.015 (0.059) | 0.45 |
| Parous | -0.54 (1.34) |  | -0.07 (0.65) |  | -0.010 (0.057) |  |
| Season of birth |  |  |  |  |  |  |
| Spring | -0.56 (1.00) | 0.131 | -0.03 (0.68) | 0.369 | -0.008 (0.054) | 0.61 |
| Summer | -0.45 (1.20) |  | 0.04 (0.50) |  | -0.016 (0.051) |  |
| Fall | -0.67 (1.50) |  | 0.05 (0.65) |  | -0.014 (0.045) |  |
| Winter | -0.78 (1.35) |  | -0.14 (0.87) |  | -0.013 (0.081) |  |

EVI: Enhanced Vegetation Index.


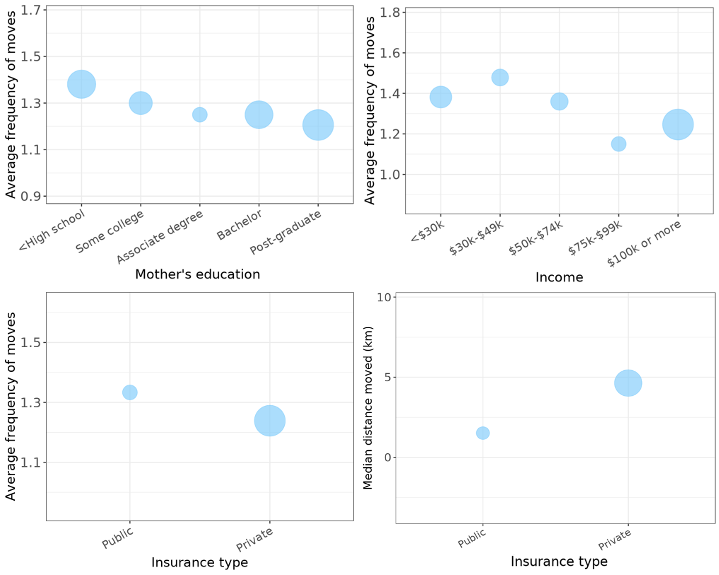


Figure S1. Frequency of moves and the total distance moved during pregnancy among the mothers who moved, by maternal factors (*n* = 417), for factors for which movers and non-movers were statistically different.


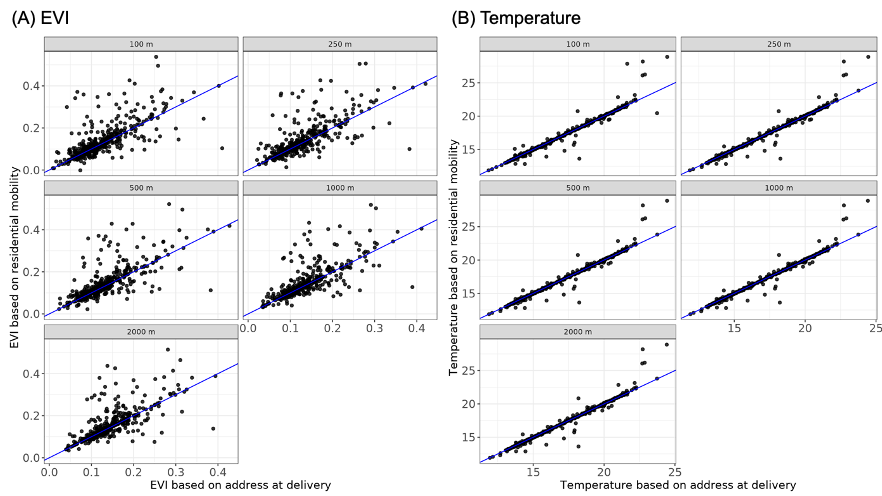


Figure S2. Exposure misclassification for EVI by buffer size around mother’s residence.

`


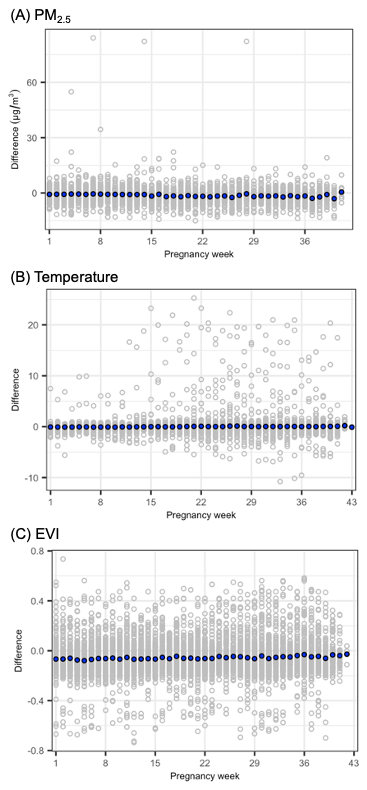


Figure S3. Exposure misclassification by gestational week (exposure based on mother’s residence minus exposure based on residential mobilities during pregnancy).


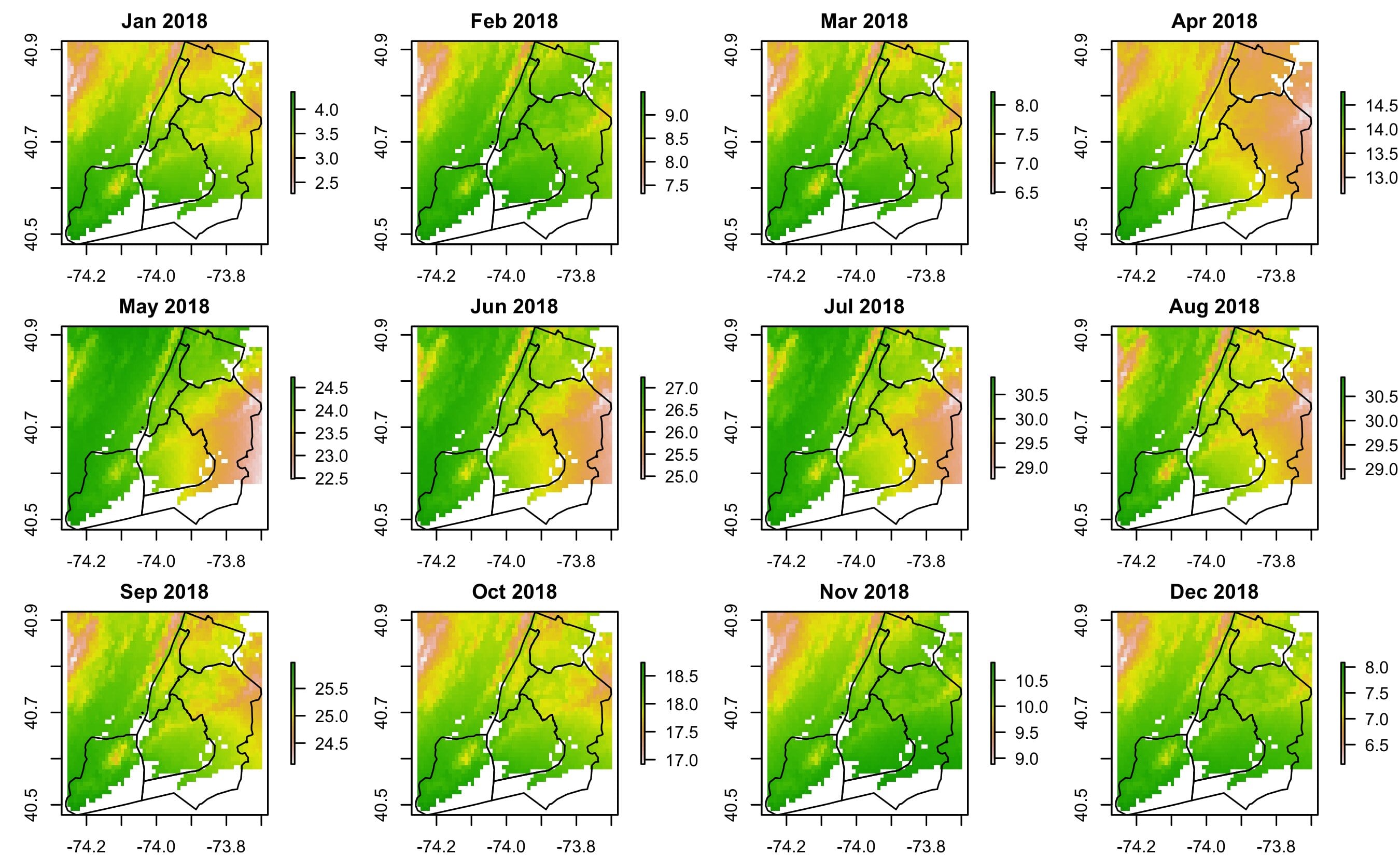


Figure S4. Map of the monthly averages of daily maximum temperature for New York City in 2018 from the Daymet.

**eAppendix - Data collection instruments**

The survey questionnaire below was used to collect data on the main analytic variables in this study. In addition, researchers extracted data from electronic health records to complete these data.

**HEALTH HISTORY**

This next set of questions is about your health when you are not pregnant.

A. In general, would you say your health is... Excellent Very good Good

Fair Poor


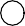

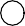

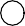

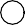

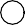


In general, would you say your quality of life Excellent

is: Very good


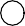

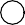

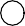

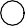

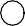


Good Fair Poor

C. In general, how would you rate your physical Excellent

health? Very good


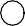

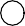

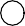

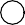

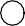


Good Fair Poor

D. In general, how would you rate your mental Excellent health, including your mood and your ability to think? Very good

Good Fair Poor


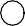

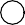

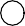

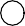

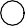


E. In general, how would you rate your satisfaction Excellent with your social activities and relationships? Very good

Good Fair Poor


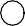

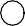

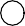

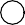

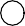


F. In general, please rate how well you carry out Excellent

your usual social activities and roles. (This includes Very good activities at home, at work and in your community, and Good responsibilities as a parent, child, spouse, employee, Fair


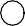

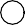

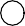

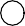

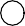


friend, etc.) Poor

G. To what extent are you able to carry out your Completely

everyday physical activities, such as walking, Mostly


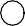

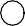

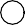

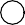

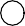


climbing stairs, carrying groceries, or moving a Moderately

chair? A little

Not at all

H. In the past 7 days, how often have you been Never

bothered by emotional problems such as feeling Rarely


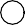

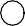

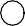

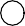

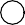


anxious, depressed, or irritable? Sometimes Often Always

I. In the past 7 days, how would you rate your None

fatigue on average? Mild


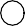

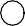

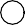

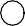

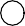


Moderate Severe Very severe

J. In the past 7 days, how would you rate your pain on average, on a scale from 0 (no pain) to 10 (worst imaginable pain)?

| **WEIGHT AND HEIGHT** |  |  |  |  |
| --- | --- | --- | --- | --- |

How tall are you without shoes? Feet/inches or meters/centimeters

What was your weight just before you became pregnant? Pounds or kilograms

| **ACTIVITY** |  |  |  |  |
| --- | --- | --- | --- | --- |

The following questions will ask you about the time you spent being physically active in the last 7 days. Please answer each question even if you do not consider yourself to be an active person. Please think about the activities you do at work, as part of your house and yard work, to get from place to place, and in your spare time for recreation, exercise or sport.

Think about all the vigorous activities that you did in the last 7 days. Vigorous physical activities refer to activities that take hard physical effort and make you breathe much harder than normal. Think only about those physical activities that you did for at least 10 minutes at a time.

During the last 7 days, on how many days did you

do vigorous physical activities like heavy lifting, digging, aerobics, or fast bicycling? (days per week)

During the last 7 days, on how many days did you

do vigorous physical activities like heavy lifting, digging, aerobics, or fast bicycling? (days per week)


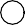
 No vigorous physical activity

How much time did you usually spend doing vigorous physical activities on one of those days?

hours per day

minutes per day


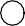
 Don't know/not sure

Think about all the moderate activities that you did in the last 7 days. Moderate activities refer to activities that take moderate physical effort and make you breathe somewhat harder than normal. Think only about those physical activities that you did for at least 10 minutes at a time.

During the last 7 days, on how many days did you do moderate physical activities like carrying light loads, bicycling at a regular pace, or doubles tennis? Do not include walking.

During the last 7 days, on how many days did you do moderate physical activities like carrying light loads, bicycling at a regular pace, or doubles tennis? (days per week) Do not include walking.


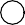
 No moderate physical activity

1. How much time did you usually spend doing moderate physical activities on one of those days?

hours per day

minutes per day


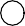
 Don't know/not sure

Think about the time you spent walking in the last 7 days. This includes at work and at home, walking to travel from place to place, and any other walking that you have done solely for recreation, sport, exercise, or leisure.

During the last 7 days, on how many days did you walk for at least 10 minutes at a time?

During the last 7 days, on how many days did you walk for at least 10 minutes at a time?(days per week)


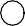
 No walking

A. How much time did you usually spend walking on one of those days?

hours per day

minutes per day


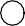
 Don't know/not sure

The last activity question is about the time you spent sitting on weekdays during the last 7 days. Include time spent at work, at home, while doing course work and during leisure time. This may include time spent sitting at a desk, visiting friends, reading, or sitting or lying down to watch television.

During the last 7 days, how much time did you usually spend sitting on a week day?

hours per day

minutes per day


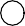
 Don't know/not sure

OUTDOORS

On average, how many hours do you spend outdoors (outside of home or work place)?

Weekdays

Weekends:

**SUBSTANCE USE**

Now we're going to ask you about various substances you may have used both before and during early pregnancy

In the 3 months before you became pregnant, did you use any of the following? Check all that apply.

Cigarettes

Cigars

Little cigars or cigarillos (e.g., Black & Milds)

Hookah/shisha/waterpipe/narghile

Electronic cigarettes, vape pens, or personal vaporizers containing nicotine (e.g., Blu, NJOY, or 'mods'). These might also include e-cigars, e-pipes, e-hookahs, and hookah pens. We are NOT including devices that vaporize pot or marijuana

Nicotine patch

Nicotine gum

Other nicotine-containing product(s) such as snuff or chewing tobacco.

I did not use any of the above

Please specify

Currently, do you use any of the following? Check all that apply.

Cigarettes

Cigars

Little cigars or cigarillos (e.g., Black & Milds)

Hookah/shisha/waterpipe/narghile

Electronic cigarettes, vape pens, or personal vaporizers containing nicotine (e.g., Blu, NJOY, or 'mods'). These might also include e-cigars, e-pipes, e-hookahs, and hookah pens. We are NOT including devices that vaporize pot or marijuana

Nicotine patch

Nicotine gum

Other nicotine-containing product(s) such as snuff or chewing tobacco.

I did not use any of the above

In the 3 months before you became pregnant, how often did you drink alcoholic beverages including wine, beer, drinks containing hard liquor, wine coolers, hard lemonade, or hard cider?

5 or more times a week

2-4 times a week

Once a week

1-3 times a month

Less than once a month

Never

How often do you currently drink alcoholic beverages?

5 or more times a week

2-4 times a week

Once a week

1-3 times a month

Less than once a month

Never

**HOUSEHOLD COMPOSITION AND DEMOGRAPHICS**

This question is about your current marital status. Are you:

Legally married

Not married but living with a partner

Single, never married

Single, separated Single, divorced Single, widowed

Do you consider yourself to be Hispanic or Latina?

Yes

No

What race do you consider yourself to be? You may select one or more.

White

Black or African American

American Indian or Alaska Native

Asian

Native Hawaiian or Other Pacific Islander

Other

What is the highest degree or level of school that you have completed?

Less than a high school diploma or GED

High school diploma or GED

Some college but no degree

Associate degree

Bachelor’s degree (e.g., Masters or Doctoral)

Now we'd like to gather some information on your partner.

Do your partner consider himself/herself to be Hispanic or Latino/Latina?

Yes

No

What race does your partner consider himself/herself to be? You may select one or more.

White

Black or African American

American Indian or Alaska Native

Asian

Native Hawaiian or Other Pacific Islander

Other

What is the highest degree or level of school that your partner has completed?

Less than a high school diploma or GED

High school diploma or GED

Some college but no degree

Associate degree

Bachelor’s degree (e.g., Masters or Doctoral)

What age is your partner?

Are you currently working at any full-time or part-time jobs?

Yes

No

**FAMILY INCOME**

Of these income groups, which category best represents your combined family income during the last calendar year?

Less than $4,999

$5,000-$9,999

$10,000-$19,999

$20,000-$29,999

$30,000-$39,999

$40,000-$49,999

$50,000-$74,999

$75,000-$99,999

$100,000-$199,000

$200,000 or more

Don't know

Were you born in the United States? Yes No

What country were you born in?

What year did you move to the United States?

What year did you move to the United States?

I did not use any of the above Cigarettes

Cigars

Little cigars or cigarillos (e.g., Black & Milds) Hookah/shisha/waterpipe/narghile

Electronic cigarettes, vape pens, or personal vaporizers containing nicotine (e.g., Blu, NJOY, or 'mods'). These might also include e-cigars,

e-pipes, e-hookahs, and hookah pens. We are NOT including devices that vaporize pot or marijuana Nicotine patch

Nicotine gum

Other nicotine-containing product(s) such as snuff or chewing tobacco.
